# Health Democracy Index: Development and Validation of a Self-Reported Instrument for Measuring Patient Participation in Health Policy

**DOI:** 10.3389/fpubh.2018.00194

**Published:** 2018-07-17

**Authors:** Kyriakos Souliotis, Lily E. Peppou, Eirini Agapidaki, Chara Tzavara

**Affiliations:** ^1^Faculty of Social and Political Sciences, University of Peloponnese, Corinth, Greece; ^2^Health Policy Institute, Maroussi, Greece; ^3^University Mental Health Research Institute, Athens, Greece; ^4^Department of Hygiene, Epidemiology and Medical Statistics, National and Kapodistrian University of Athens, Athens, Greece

**Keywords:** scale development, patient participation, patient empowerment, patient organizations, health policy decision making

## Abstract

**Introduction:** Patient participation has emerged as a preponderant theme in contemporary health and healthcare; however there is a dearth of research on the degree and impact of collective patient participation on shaping health policy. In this frame, the current study endeavored to validate a scale for assessing patients' association (PA) participation in health policy processes. Furthermore, PAs' participation in health policy decision making in Greece was explored.

**Materials and Methods:** The Health Democracy Index (HDI) is an eight-item scale enquiring about PAs' participation in important facets of health policy. To investigate its psychometric properties, 414 members of PAs in Greece were randomly recruited. By employing a self-reported questionnaire, construct validity was examined through exploratory and confirmatory factor analysis, while convergent validity was investigated through an additional question asking respondents to rate the degree of their association's participation in health policy processes. Moreover, the internal consistency of the scale and its test-retest reliability were explored.

**Results:** The scale showed high internal consistency (Cronbach a = 0.85) and test-retest reliability (ICC = 0.89, *p* < 0,001). Exploratory factor analysis suggested a unidimensional construct; while confirmatory factor analysis indicated an adequate fit of the one-factor model (RMSEA = 0.079, CFI = 0.976, and GFI = 0.972). Regarding convergent validity, the HDI composite score displayed strong and positive correlation with the item asking respondents to rate the degree of PA participation in health policy processes (rho = 0.73, *p* < 0.0001). Concerning the pattern of results in Greece, PAs' participation was found to be low. The lowest level was observed for the item enquiring about PA participation in the national parliament and the highest for panels at influential health-related organizations.

**Conclusion:** The HDI is a valid and reliable tool that can be utilized to serve policy-related as well as research purposes. PAs' participation in Greece is weak and thus efforts should be made to enhance it.

## Introduction

Patient participation is a multidimensional concept that emerged between late 1960s and early 1970s so as to describe patients' rights to be aware of the options, risks, and benefits of treatment as well as to make informed choices (1–3). The difficulty in defining patient participation reflects the diversity of its applicability (4, 5) as its conceptualization is often bound to the context in which it occurs (6, 7). In this rationale, many researchers have delineated patient participation as the process that enables patients to play an essential part in the decision making that affects their health; for example, in shared-decision making upon developing a treatment plan (8, 9).

However, in contemporary health care systems patients not only participate in decisions regarding their own health and treatment (i.e., micro-level), but in decision-making processes at the meso- and macro-level—i.e., in local health authorities, organizations or at the national level (10–12). In particular, patients participate in decision-making in various realms, such as government policy, medical guideline development, health technology assessment and biomedical research, among others (12–16). Patients' involvement in these processes is justified by their experiential knowledge, which is posited to improve the quality of decisions (17). Congruent with this, the overall effectiveness and efficiency of health care systems may rise, as patients might suggest solutions consonant with their preferences, thereby averting errors and saving costs (18–20). Patient participation is also justified on democratic grounds, as people who attain benefits or face the consequences of noteworthy decisions ought to play a part in the process. At the same time, participation in policy processes may also constitute a vehicle for patient empowerment, as it may enhance their sense of self efficacy (12, 17, 21). A growing body of research has corroborated a positive influence of patient participation on quality of care, the performance of health care services and population health outcomes, across a range of illnesses (20, 22, 23).

The elevated prevalence of non-communicable diseases worldwide has led to increased demands and costs resulting from the substantial influence of chronic illnesses on health and healthcare (24, 25). Many programs and interventions have been designed and conducted internationally so as to increase patient awareness and participation. These initiatives have endeavored to improve health literacy, enhance patient engagement in clinical decision making and promote self-management (10, 26–28).

Programs at the meso- or macro- level are rare and primarily fulfill advocacy goals. Many countries in Europe have established pertinent legislation, while they have launched public campaigns in order to raise awareness and facilitate patient empowerment. The majority of countries have founded governmental and nongovernmental organizations and taken a “bottom-up” approach to foster patient participation in health decision making (23). Specifically, countries such as Australia, Canada, the Netherlands, Germany and the United Kingdom have founded federal and regional health councils and inaugurated initiatives that would assign patients a pivotal role in health policy decision making (23, 29–32). In the United States of America, participatory decision making models have been promoted in order to advance patient participation and provide individuals with ample opportunity to engage with policy makers so as to contribute substantially in formulating legislation and health interventions (33, 34). A similar action in France resulted in a bill that fostered the creation of a more democratic atmosphere in health (“démocratie sanitaire”) and healthcare through patient participation (35).

Although there are some instruments that assess the degree of patient involvement in decision making in the micro-level, there are no validated tools for evaluating the impact of patient participation at meso-or macro-level (36). Therefore, any initiative, legislation or policy aiming to induce change at these levels cannot be consistently and systematically evaluated. Moreover, any empirical study conducted along these lines is heavily based on qualitative methodology (12, 37, 38). Thus, the development and validation of an index measuring patient participation at the meso and macro-level can be a way forward for reconciling the patient-centered paradigm and the evidence-based paradigm in health and health care. This integration has been described as the future challenge for researchers, policy makers and other stakeholders (39).

In Greece, the Legislative Act No1397/1983, which enabled the foundation of the National Health System, provided the establishment of regional health boards, where the local community would have the opportunity to be involved in the germane processes. However, this was never put into practice (40, 41). Any initiative undertaken by patients to participate in healthcare reforms has not been consistent or systematic, since there has been no legal framework or political culture to foster such a development (22). The last few years patient associations (PAs) in Greece have extended their activities from advocacy to claiming their involvement in health policy processes (22). In spite of their efforts, the National Health System remains largely centralized with respect to the organization and administration of health services. Regional health authorities or other pertinent bodies are constrained into having an advisory and often imperfect supervisory role without exerting any real influence on decision-making processes (42). This centralist approach leaves little room for public involvement in health care.

In this context, the current study aims to describe the development of an original instrument assessing PAs' participation at the meso and macro-level and to explore its psychometric properties. Moreover, by utilizing this tool, PAs' participation in health policy decision-making in Greece is delineated.

## Methods

### Instrument development

The Health Democracy Index (HDI) is an original scale consisting of 8 items, gleaning information about PAs' participation in health policy decision processes at the meso- and macro-level: reforms, panels at the Ministry of Health, panels in other influential health-related organizations, hospital boards, Ethics Committees in clinical trials, Health Technology Assessment procedures and the national parliament; from the patient perspective. For each question, 7 rating options were available: (i) it is not a legal requirement and it never happens, (ii) it is not a legal requirement and it rarely happens, (iii) it is not a legal requirement but it often happens, (iv) it is a legal requirement and it never happens, (v) it is a legal requirement and it often happens, (vi) it is a legal requirement and it happens very often, and (vii) it is a legal requirement and it always happens. In addition, there was one question enquiring about the frequency whereby a substantial change in a health policy decision was evoked as a result of PA's involvement. For this question, ratings were made on a 7-point scale tapping a frequency dimension: never-very rarely-rarely-sometimes-often-very often-always.

For developing the scale, the following procedure was undergone: (i) definition of the construct, (ii) review of the construct definition, (iii) item drafting, (iv) item review, (v) pilot testing of its psychometric properties. In terms of reliability, internal consistency— i.e., the degree to which the items of a scale tap a common underlying construct- as well as test-retest reliability– i.e., the stability of a scale over time- were explored. With respect to validity, construct validity—the degree to which the scale properly addresses a real, coherent and meaningful concept- and convergent validity—the degree to which the scale is correlated as predicted with another measure tapping the same or a similar construct—were investigated.

In particular, after PA's participation in health policy processes was defined, its definition was reviewed by 34 stakeholders in the field: patients-members of PAs (*n* = 10), representatives of PAs (*n* = 10), health policy makers (*n* = 5), healthcare providers (*n* = 5), and researchers (*n* = 5). Purposive sampling was employed so as to select the members of the panel, with their level of knowledge and relevance of experience being the main inclusion criteria.

Item drafting entailed compiling a list of potential items, derived from existing international literature e.g., (11, 12, 38). Moreover, 12 patients, who were active members of PAs, took part in a focus group addressing PA participation in health policy decision making. An email was sent to all PAs explaining to them the purpose of the focus group and inviting them to send one of their members to participate. It is noteworthy that this member should not have taken part in any other aspect of the study (i.e., the expert panel). The focus group occurred in Athens, it was facilitated by two researchers of our team (Lily Peppou and Eirini Agapidaki), with experience on qualitative methods, and it lasted 1 h and a half. The topic guide consisted of open-ended questions on PA's participation on the meso- and macro-level, its various aspects and its strengths and difficulties. The content of the focus group was audio taped and transcribed verbatim; while the data were analyzed by the two researchers independently by employing thematic analysis. It is noteworthy that inter-rater reliability was deemed high (range of Cohen's kappa coefficient for emerging themes 0.78–0.84). As a result of this, the final version of the scale consisted of 10 items.

The experts who reviewed the construct definition were invited for a second time so as to provide their feedback on the final items. Consensus was reached upon the first round and the 10 items were reduced into 8. The item “Does your PA participate in steering committees for clinician guideline development?” was suggested to be included into the item enquiring about PA's participation in panels or workshops in influential health-related organizations—such as the National Organization for Healthcare Provision or the Regional Health Directorate-, along with “participation in the National Organization for Medicines.” Furthermore, the experts suggested weighting scale items, in round 2, as participation in these different realms of helth policy decision making are not of equal prominence. The revised questionnaire was sent back for weights to be assigned to each item of the index. More specifically, the weighting was done in accord with the Rank Order Centroid Method—a statistical technique employed to generate a modeled relative ranking among various criteria—, as it has been argued that decision makers can usually rank items much more easily than assign weights to them. Therefore, the construct and content validity— i.e., the degree to which the items are appropriate in addressing the construct of interest—of the scale were bolstered.

The HDI was distributed to participants of the present study in order to investigate further its psychometric properties. In addition, the questionnaire of the present study encompassed questions enquiring about the socio-demographic characteristics of respondents, their illness, and one question enquiring about PA's degree of participation in health policy decision processes. Rating options for this question ranged from 1 to 6 (1 = absent and 6 = very high). The particular question was entailed in the questionnaire, so as to examine the convergent validity of the index.

### Sample and data collection

The sample of the present study consisted of members of PAs located in Athens area, who have not previously participated in the other steps of the index development (i.e., expert panel and focus group). As the health care system in Greece is centralized, PAs are clustered around Athens region, where the vast majority of decision-making bodies for shaping health policies are situated (e.g., the Ministry of Health, the Greek parliament, Hospitals boards and Advisory boards, among others). In this way, they can promote their involvement in activities and decision-making processes. Nonetheless, as the members of these PAs reside in all parts of Greece, the sample of the present study was deemed nationwide.

To be approached for participation in the study, PA was defined as any non-profit organization with a legal entity, primarily composed of patients and their caregivers, that represents and/or supports their needs (43). Moreover, the disease of reference should have been that of a chronic illness. A list of PAs was attained by the Ministry of Health, complemented by an Internet search. In total 20 PAs were found to meet the criteria for participation. An email was sent to the board members of all PAs inviting them to participate in the study. All PAs were interested in participating and thus the institutional review board of each PA approved the study protocol. A random sample of members from each PA was selected to take part in the interview. The required size of the sample was computed by employing the subject to item ratio, the most frequently used sample size calculation method for scale validation (44). To be eligible for participation, members of PAs had to be older than 18 years old and be fluent in Greek language. In each PA, its board members sent the invitation for participation, information about the study and the written informed consent via email or fax to all of its members. Once a member signed the written consent, the questionnaire was sent to him or her via email, fax or a web-link. Out of the 553 people invited to participate, 440 completed the self-reported instrument (response rate = 74.9%). According to Comrey and Lee (45), a sample size of 400–500 is deemed very good for scale development and validation. Moreover, for a random sub-sample of 100 participants, 1 week after the initial administration, the instrument was completed for the second time in order to assess its test-retest reliability.

### Ethics, consent and permission

Research was approved by the Research and Ethics Committee of the University of Peloponnese, in line with the ethical standards set forth in the 1964 Declaration of Helsinki. Additionally, the Institutional Review Board of the PAs which agreed to participate in the study discussed and approved the research protocol. Informed consent for participation was gleaned from all participants.

### Statistical analysis

Continuous variables are indicated with mean and standard deviation (SD). Nominal variables are indicated with absolute and relative frequencies. The sample was randomly split into two datasets of approximately equal size. Data of the even subsample (*N* = 217) were used to perform an exploratory factor analysis (EFA) so as to examine the construct validity of the index and to ascertain whether the scale could be regarded as a measure of a single construct. Principal component analysis (PCA) was selected as extraction method using Varimax rotation. The cut-off point for factor loadings was 0.40 and for eigenvalues it was 1.00. A confirmatory factor analysis (CFA) with maximum likelihood procedure was conducted on the odd subsample (*N* = 197) so as to confirm the model emerging from the EFA. The variance of the latent constructs was fixed at one during parameter estimation. The fit of the CFA model was tested using the chi square (χ^2^), the comparative fit index (CFI), the goodness of fit index (GFI) and the root mean square error of approximation (RMSEA) (46). For the CFI and GFI indices, values close to or greater than 0.95 are considered to indicate a good fit to the data (47). RMSEA values of less than 0.05 indicate a good fit and values as high as 0.08 indicate a reasonable fit (46). In addition, a non significant chi square statistic suggests a good fit, but chi square is readily influenced by sample sizes and thereby significant for large sample sizes (46).

The internal consistency of the scale was examined with Cronbach's α; while test-retest reliability with intra-class correlation coefficient. Reliability equal to or greater than 0.70 was deemed appropriate. Spearman correlation coefficients were computed to investigate the association between the HDI dimensions score and the degree of participation of their organization in health policy decision making. Correlation coefficient between 0.1 and 0.3 is regarded low, between 0.31 and 0.5 moderate and over 0.5 high. *P*-values reported are two-tailed. Statistical significant level was set at 0.05 and analysis was carried out by using SPSS and AMOS (SPSS, Chicago, IL, USA) Statistical Software.

## Results

### Sample characteristics

Participants were 154 men and 260 women (*N* = 414) with mean age 44.9 years (SD = 12.3 years). Most of the participants were Greek (99.5%), and more than half of them (63.5%) had more than twelve educational years. Sample characteristics are presented in Table 1.

**Table 1:** Sample characteristics.

	***N* (%)**
Age, mean (SD)	44.9 (12.3)
**SEX**	
Men	154 (37.2)
Women	260 (62.8)
**MARITAL STATUS**	
Married/civil partnership/Co-habiting	214 (53)
Widowed	18 (4.4)
Divorced	40 (9.9)
Single/non co-habiting partner	132 (32.7)
**NATIONALITY**	
Greek	396 (99.5)
Other	2 (0.5)
**EDUCATIONAL LEVEL**	
< 12 years of education	48 (11.9)
12 years of education	152 (37.8)
12 < years of education	202 (50.2)
**POSITION IN THE PA**	
President or other board members	100 (24.8)
Voting member	198 (49.0)
Active but non-voting member	8 (2.0)
Non-active and non-voting member	94 (23.3)
Other	4 (1.0)

### Descriptive statistics for the HDI

Descriptive statistics for the HDI items are illustrated in Table 2. The higher median value was 4 and was found for the question “How often do you observe a substantial change in the content of a health policy decision as a result of interference from a patient organization?,” while the lowest median value was 1 and it was found for the questions “Does your PA take part in boards of hospitals?” and “Does your PA is present in the national parliament during decision making for important health policies/issues?”

**Table 2:** Descriptive statistics for the HDI items.

	**Mean (SD)**	**Median (IQR)**
Does your patient organization take part in reforms or crucial decisions in health policy?	3.4 (1.8)	3 (2–5)
Does your patient organization take part in workshops or panels held at the Ministry of Health?	3.4 (1.9)	3 (2–5)
Does your patient organization take part in workshops or panels in other important organizations, pertinent to health?	3.6 (1.7)	4 (2–5)
Does your patient organization take part in boards of hospitals?	1.9 (1.4)	1 (1–2)
Does your patient organization take part in Ethics Committees for clinical trials?	2.1 (1.3)	2 (1–3)
Does your patient organization take part in Health Technology Assessment procedures?	2.3 (1.6)	2 (1–3)
Does your patient organization is present in the national parliament during decision making for important health policies/issues?	1.8 (1.2)	1 (1–2)
How often do you observe a substantial change in the content of a health policy decision as a result of interference from a patient organization? (yours or another's)	3.7 (1.6)	4 (2–5)
*HDI total score*	22.2 (8.6)	22 (16–28)

### Construct validity EFA, CFA results, internal consistency and test-retest reliability

A principal components analysis with a varimax rotation was used the even subsample. Items loadings as derived from factor analysis are depicted in Table 3. In the final model all items were entered into the factor analysis. One factor emerged with eigenvalue more than one and accounted for 48.7% of the total variance. Factor loadings ranged from 0.55 (for the item “Does your patient organization is present in the national parliament during decision making for important health policies/issues?”) to 0.81 (for the item “Does your patient organization take part in reforms or crucial decisions in health policy?”). Inter-item correlation coefficients and Cronbach's a if an item was deleted are presented in Table 3. Internal consistency reliability for the HDI was good with Cronbach's alpha equal to 0.85. The mean inter-item correlation was 0.41. Subsequently, a CFA was performed in the odd subsample to examine whether the one-dimensional model fitted the data well. The CFA corroborated an adequate fit of the one-factor model (RMSEA = 0.079, CFI = 0.976, and GFI = 0.972). None of the item cross loadings exceeded the item loadings on the intended latent construct. The chi-square test of the model was significant (*p* < 0.05). Test-retest reliability was high (ICC = 0.89, *p* < 0.001).

**Table 3:** Factor loadings form the results of exploratory factor analysis, inter-item correlation coefficients and internal consistency reliability of the HDI questionnaire.

	**Factor loading**	**Inter-item correlation coefficients**	**Cronbach's a if item deleted**
Does your patient organization take part in reforms or crucial decisions in health policy?	0.81	0.72	0.81
Does your patient organization take part in workshops or panels held at the Ministry of Health?	0.78	0.68	0.82
Does your patient organization take part in workshops or panels in other important organizations, pertinent to health?	0.78	0.69	0.82
Does your patient organization take part in boards of hospitals?	0.59	0.46	0.84
Does your patient organization take part in Ethics Committees for clinical trials?	0.66	0.54	0.84
Does your patient organization take part in Health Technology Assessment procedures?	0.76	0.65	0.82
Does your patient organization is present in the national parliament during decision making for important health policies/issues?	0.55	0.44	0.85
How often do you observe a substantial change in the content of a health policy decision as a result of interference from a patient organization? (yours or another's)	0.61	0.50	0.84

### Convergent validity

Table 4 depicts correlation coefficients of the HDI items with the degree of PA participation in health policy decision making. All HDI items were positively and significantly correlated with the responses concerning the degree of PA participation in health policy decision making. The highest correlations were found with the items “How often do you observe a substantial change in the content of a health policy decision as a result of interference from a patient organization?,” “Does your patient organization take part in reforms or crucial decisions in health policy?” and “Does your patient organization take part in workshops or panels held at the Ministry of Health?”

**Table 4:** Correlation coefficients of the HDI items with the degree of participation of their organization in health policy decision making.

	**Degree of PA participation in health policy decision making**
Does your patient organization take part in reforms or crucial decisions in health policy?	0.65
Does your patient organization take part in workshops or panels held at the Ministry of Health?	0.57
Does your patient organization take part in workshops or panels in other important organizations, pertinent to health?	0.53
Does your patient organization take part in boards of hospitals?	0.19
Does your patient organization take part in Ethics Committees for clinical trials?	0.33
Does your patient organization take part in Health Technology Assessment procedures?	0.54
Does your patient organization is present in the national parliament during decision making for important health policies/issues?	0.30
How often do you observe a substantial change in the content of a health policy decision as a result of interference from a patient organization? (yours or another's)	0.68
HDI total score	0.73

*All correlations were significant at p < 0.001*.

The total score of HDI was highly and positively correlated with responses on the question about how they rate the degree of PA participation in health policy decision making (rho = 0.73, *p* < 0.001), indicating good convergent validity.

## Discussion

The present study elaborated on the development of an original index for measuring PA's participation in health policy decision-making processes from the patient standpoint. It described the steps of its development and provided evidence for its psychometric properties. At the same time, due to the nationwide sample of participants recruited, the present study also gives a snapshot of PAs' participation in health policy in Greece.

Concerning the psychometric characteristics of the scale, it displayed good construct, face and convergent validity; while it also demonstrated high internal consistency and inter-rater reliability. The index was found to measure PA's degree of participation in health policy decision-making processes and in accord to the factor analysis output the construct was found to be unidimensional. The particular index fills a gap in the existing literature, as the instruments that have been developed to measure patient involvement in decision-making usually address individual patients and their relationships with their physicians (36). Nonetheless, even these instruments that are postulated to assess patient participation in the micro-level, are often shown to be not well developed or validated (36). A review article on the topic concludes that any valid instrumentation should stem from a well-defined construct with item drafting on the grounds of qualitative inquiry and then rigorously developed by adhering to psychometric principles (36). The HDI development has followed exactly this approach and therefore it constitutes a novelty in the international literature.

Concomitantly, it has been posited that the past few years, the two preponderant paradigms in health and healthcare— i.e., the evidence-based paradigm and the patient-centered one— seem to originate from two different worlds, which appear incompatible at first glance (39). The challenge for policy makers, researchers, patients, and healthcare professionals in the foreseeable future is to find ways to integrate them. Congruent with this, the HDI is an effort to do so. Specifically, there is only a handful of studies exploring PA's participation in health policy at meso- or macro-level and primarily emanate from UK, US, and the Netherlands (12, 37, 38, 48). These studies adhere largely to the patient-centered paradigm and hence employ qualitative methodology. At the same time, existing controversy with regard to the involvement of PAs in health policy decision—that is whether in practice it constitutes mere tokenism (49)—is not addressed by rigorous methodology and it is often based on reasonable arguments and commentaries. Furthermore, particular hypotheses about the positive correlates of PAs' participation in health policy cannot be addressed adequately with existing methods. For example, there are two conflicting views with regard to the influence of the decentralization of health care systems on PA's participation in health policy. On the one hand, it may provide ample opportunity for PAs to engage with decision makers and shape policy. On the other hand, it may weaken their operation by diluting their capacity and diffusing their resources (38). So far, no empirical study has verified/refuted these hypotheses.

In this frame, a quantitative instrument that would complement existing qualitative methods, can shed light on these processes and elucidate their influences. Moreover, the HDI can be used to facilitate comparisons among countries or to monitor changes across time within the same country. Given its high test-retest reliability, it can also be utilized to gauge the effectiveness of legislation, policies, programs and interventions targeting patient participation in the meso- and macro- level. It can be used to explore specific hypotheses and to generate new ones, advancing therefore our knowledge on the field. Furthermore, it can inform advocacy initiatives by providing recommendations for promoting and optimizing PA participation in health policy decision-making processes. Arguably, it can aid the development of a roadmap, entailing the necessary steps for PAs to acquire a central position in the decision-making processes in their respective countries as well as in Europe. It is noteworthy that since the instrument taps the patient perspective on PA participation in health policy decision making, a content analysis of relevant documents (e.g., meeting minutes of national parliament, legal documents, etc.) would complement this research approach, illuminating further these processes.

Concerning Greece, the long standing observation that PAs do not exert substantial influence on health policy (22, 42) is largely verified by present data. The median values for all the items of the HDI were below the mid-point, with lowest participation being observed with regard to PAs' presence in the parliament during discussions for pivotal health decisions and the highest in PA's participation in panels in influential health-related organizations. Interestingly, the median value for the item “How often do you observe a substantial change in the content of a health policy decision as a result of interference from a PA?” was high as well. This finding appears contradictory at first glance, as how can PAs have limited participation in important decision-making bodies and at the same time feel that a noteworthy change occurs in the content of a health policy decision due to interference from PAs? This finding can explained by the joint influence of advocacy and PAs' efforts in changing health policy decisions, since the wording of the item leaves room for change to be elicited by the respondents' PA or by other PAs. Alternatively, it may reflect respondents' ignorance, as any change that might have occurred is erroneously ascribed to the workings of another PA. In spite of the diversity in interpretations for the particular item, it is noteworthy that its statistical properties (factor loading and inter-item correlation coefficient) were adequate. A future research report will elaborate on the correlates of PAs participation in health policy process in Greece.

In spite of the novelty of the study, it was not without its limitations. The scale development occurred on a Greek sample and therefore its psychometric properties may not apply to other settings. The rationale for limiting respondents to Greek members of PAs pertains to an endeavor to have the sample as homogenous as possible in order to facilitate psychometric exploration. Moreover, as the results of the validation process could not have been anticipated, recruiting PAs from other countries was thwarted by feasibility issues (no sampling frame of PAs in Europe, difficulty in setting criteria for selecting only a handful of European countries, time-consuming study, self-funded research, etc.). Arguably, the aspects of PAs' participation included in the HDI are international and are thus of relevance to other countries as well; however, a further exploration of its psychometric properties in other countries will enhance its robustness. The authors of the present study consider instrument validation to be a long and ongoing process and therefore the present manuscript is regarded as the first step in the validation process of the HDI. It merits noting that our research team is currently investigating the psychometric properties of the HDI in Cyprus, Italy and France. Moreover, the scale taps the patient perspective on PA participation in health policy decision making and therefore it does not substitute nor it cancels out other perspectives (e.g., policy makers' perspective or evidence emanating from document analysis). These perspectives should be taken into consideration jointly.

## Conclusions

Findings of the present study substantiate the psychometric properties (construct validity, face validity and convergent validity; internal consistency and test-retest reliability) of the Health Democracy Index, an original scale for assessing PA's participation in health policy decision-making. Furthermore, by administering the particular instrument to a random and representative sample of patients-members of PAs in Greece, it was found that PAs' participation in Greece is low and efforts should be made to enhance it.

## Author contributions

KS: conception and design of research, data interpretation, critically revising the manuscript for important intellectual content. LP: design of the work, data interpretation, drafting the work. EA: design of the work, data interpretation, drafting the work. CT: design of the work, data analysis, drafting the work.

### Conflict of interest statement

The authors declare that the research was conducted in the absence of any commercial or financial relationships that could be construed as a potential conflict of interest. The reviewer MS and handling Editor declared their shared affiliation.

## References

[1] Arrow K Uncertainty and the welfare economics of medical care. Am Econ Rev. (1963) 53:941–73.

[2] Bambra C Fox D Scott-Samuel A. Towards a politics of health. Health Promot Int. (2005) 20:187–93. 10.1093/heapro/dah608 15722364

[3] World Health Organization Primary Health Care, Alma Ata. Geneva: WHO (1978).

[4] Cahill J. Patient participation–a review of the literature. J Clin Nurs. (1998) 7:119–28. 10.1111/j.1365-2702.1998.00132.x 9582762

[5] Entwistle VA Watt IS. Patient involvement in treatment decision-making: the case for a broader conceptual framework. Patient Educ Couns. (2006) 63:268–78. 10.1016/j.pec.2006.05.002 16875797

[6] Bruegel R. Patient empowerment–a trend that matters. J AHIMA (1998) 69:30–3. 10182504

[7] Thompson AG. The meaning of patient involvement and participation in health care consultations: a taxonomy. Soc Sci Med. (2007) 64:1297–310. 10.1016/j.socscimed.2006.11.002 17174016

[8] Tattersall RL. The expert patient: a new approach to chronic disease management for the twenty-first century. Clin Med. (2002) 2:227–9. 10.7861/clinmedicine.2-3-227 12108472 PMC4954037

[9] Dicker A Armstrong D. Patients' views of priority setting in health care: an interview survey in one practice. BMJ (1995) 311:1137–9. 7580710 10.1136/bmj.311.7013.1137 PMC2551058

[10] Coulter A Parsons SJA Where are the Patients in Decision-Making About Their Own Care? Health Systems and Policy Analysis. Copenhagen: WHO (2008).

[11] Baggott R Allsop J Jones K Speaking for Patient and Carers: Health Consumer Groups and the Policy Process. Basingstoke: Palgrave Macmillan (2005).

[12] Van de Bovenkamp HM Trappenburg MJ Grit K. Patient participation in collective health care decision-making: the Dutch model. Health Expect. (2010) 13:73–85. 10.1111/j.1369-7625.2009.00567.x 19719537 PMC5060518

[13] Crawford MJ Rutter D Manley C Weaver T Bhui K Fulop N. Systematic review of involving patients in the planning and development of health care. BMJ (2002) 325:1263. 10.1136/bmj.325.7375.1263 12458240 PMC136920

[14] Ross EC Report from the field regulating managed care: interested group competition for control and behavioral health care. J Health Polit Policy Law (1999) 24:599–625. 10.1215/03616878-24-3-599 10386328

[15] Menon D Stafinski T. Role of patient and public health technology assessment and coverage decisions. Expert Rev Pharmacoecon Outcomes Res. (2011) 11:75–89. 10.1586/erp.10.82 21351860

[16] Abma TA Broerse JE. Patient participation as dialogue: setting research agendas. Health Expect. (2010) 13:160–73. 10.1111/j.1369-7625.2009.00549.x 20536537 PMC5060528

[17] Caron-Flinterman JF Broerse JE Bunders JF. The experiential knowledge of patients: a new resource for biomedical research? Soc Sci Med. (2005) 60:257–84. 10.1016/j.socscimed.2004.11.023 15814182

[18] Boote JD Telford R Cooper CL. Consumer involvement in health research: a review and research agenda. Health Policy (2002) 61:213–36. 10.1016/S0168-8510(01)00214-7 12088893

[19] Epstein S Patient groups and health movements. In: Hackett EJ, Amsterdamska O, Lynch M, Wajcman J, editors. The Handbook of Science and Technology Studies. Massachusetts: MIT Press (2008). pp. 499–539.

[20] World Health Organization Exploring Patient Participation in Reducing Health-Care-Related Safety Risks. Copenhagen: WHO (2013).

[21] Baker A. Patient involvement in a professional body: reflections and commentary. J Health Organ Manag. (2007) 21:460–9. 10.1108/14777260710778970 17933376

[22] Souliotis K Looking for democracy in health amidst the fiscal crisis: patient participation in health policy decision making. In: Souliotis K, editor, Democracy, Citizens and Health Policy: Participation in Decision Making, Lobbying and Patient Associations (in Greek). Athens: Papazisis (2014). pp. 23–51.

[23] Conklin A Morris Z Nolte E Involving the Public in Healthcare Policy: An Update of Research Evidence and Proposed Evaluation Framework. A Technical Report. Cambridge: RAND Europe (2010).

[24] Hoglund AT Winblad U Arnetz B Arnetz JE. Patient participation during hospitalization for myocardial infarction: perceptions among patients and personnel. Scand J Caring Sci. (2010) 24:482–9. 10.1111/j.1471-6712.2009.00738.x 20230518

[25] Weingart SN Zhu J Chiappetta L Stuver SO Schneider EC Epstein AM. Hospitalized patients' participation and its impact on quality of care and patient safety. Int J Qual Health Care (2011) 23:269–77. 10.1093/intqhc/mzr002 21307118 PMC3140261

[26] Heisler M Bouknight RR Hayward RA Smith DM Kerr EA. The relative importance of physician communication, participatory decision making, and patient understanding in diabetes self-management. J Gen Intern Med. (2002) 17:243–52. 10.1046/j.1525-1497.2002.10905.x 11972720 PMC1495033

[27] Holman H Lorig K. Patients as partners in managing chronic disease. BMJ (2000) 320:526–7. 10.1136/bmj.320.7234.526 10688539 PMC1117581

[28] Nutbeam D Health literacy as a public health goal: a challenge for contemporary health education and communication strategies into the 21st century. Health Promot Int. (2000) 15:259–67. 10.1093/heapro/15.3.259

[29] Eliasoph H Monaghan B Beaudoin R. We are all in this together: integrated health service plans in Ontario. Healthc Q. (2007) 10:82–7. 10.12927/hcq.2007.18938 18271108

[30] Wiseman V Mooney G Berry G Tang KC. Involving the general public in priority setting: experiences from Australia. Soc Sci Med. (2003) 56:1001–12. 10.1016/S0277-9536(02)00091-6 12593873

[31] Oliver S Clarke-Jones L Rees R Milne R Buchanan P Gabbay J. Involving consumers in research and development agenda setting for the NHS: developing an evidence-based approach. Health Technol Assess. (2004) 8:1–148. 10.3310/hta8150 15080866

[32] Legare F Stacey D Forest PG. Shared decision-making in Canada: update, challenges and where next! Z Arztl Fortbild Qualitatssich (2007) 101:213–21. 10.1016/j.zgesun.2007.02.024 17601175

[33] Goold SD Biddle AK Klipp G Hall CN Danis M. Choosing healthplans all together: a deliberative exercise for allocating limited health care resources. J Health Polit Policy Law (2005) 30:563–601. 10.1215/03616878-30-4-563 16318163

[34] Sheridan SL Harris RP Woolf SH. Shared decision making about screening and chemoprevention: a suggested approach from the U.S. preventive services task force. Am J Prev Med. (2004) 26:56–66. 10.1016/j.amepre.2003.09.011 14700714

[35] Vega A Healthcare democracy and society's challenges with regard to medicines. Soins (2014) 784:31–3. 10.1016/j.soin.2014.02.003 24839681

[36] Elwyn G Edwards A Mowle S Wensing M Wilkinson C Kinnersley P. Measuring the involvement of patients in shared decision-making: a systematic review of instruments. Patient Educ Couns. (2001) 43:5–22. 10.1016/S0738-3991(00)00149-X 11311834

[37] Tyler S. Comparing the campaigning profile of maternity user groups in Europe–can we learn anything useful? Health Expect. (2002) 5:136–47. 10.1046/j.1369-6513.2002.00172.x 12031054 PMC5060145

[38] Baggott R Forster R. Health consumer and patients' organizations in Europe: towards a comparative analysis. Health Expect. (2008) 11:85–94. 10.1111/j.1369-7625.2007.00472.x 18275405 PMC5060431

[39] Bensing J Bridging the gap. the separate worlds of evidence based medicine and patient centred medicine. Patient Educ Couns. (2000) 39:17–25. 10.1016/S0738-3991(99)00087-7 11013544

[40] Theodorou M Samara K Pavlakis A Middleton N Polyzos N Maniadakis N The public's and doctor's perceived role in participation in setting health care priorities in Greece. Hellenic J Cardiol. (2010) 51:200–8. 20515851

[41] Kolovos P Sourtzi P The concept of participation in healthcare (in Greek). Hellenic J Nurs. (2007) 46:38–47.

[42] Kakaletsis N Ioannidis A Sigalas I Hatzitolios A The regional organization of the National Health System (ESY) in Greece–a brief overview of the legislative interventions to date. Arch Hellenic Med. (2013) 30:233–40.

[43] European Federation of Pharmaceutical Industries and Associations EFPIA Code of Practice on Relationship Between the Pharmaceutical Industry and Patient Organizations. (2011). Available online at: http://transparency.efpia.eu/uploads/Modules/Documents/code_po(2011).pdf

[44] Anthoine E Moret L Regnault A Sebille V Hardouin JB. Sample size used to validate a scale: a review of publications on newly-developed patient reported outcome measures. Health Qual Life Outcomes (2014) 12:2. 10.1186/s12955-014-0176-2 25492701 PMC4275948

[45] Comrey AL Lee HB A First Course in Factor Analysis. Hillisdale, NJ: Lawrence Erlbaum Associates (1992). p. 488.

[46] Mueller R Basic Principles of Structural Equation Modeling. New York, NY: Springer (2000).

[47] Hu L Bentler P Cutoff criteria for fit indices in covariance structure analysis: convetnional criteria versus new alternatives. Struct Equ Model. (1999) 6:1–55. 10.1080/10705519909540118

[48] Wood B Patient Power: the Poltics of Patients' Associations in Britain and America. Buckingham: Open University Press (1999).

[49] Martin GP. Citizens, publics, others and their role in participatory processes: a commentary on Lehoux, Daudelin and Abelson. Soc Sci Med. (2012) 74:1851–3. 10.1016/j.socscimed.2012.02.030 22503560

